# Analysis of the Synergistic Effect on the Strength Characteristics of Modified Red Mud-Based Stabilized Soil

**DOI:** 10.3390/ma16186104

**Published:** 2023-09-07

**Authors:** Shengjin Chen, Jie Jiang, Xiaoduo Ou, Zhijie Tan

**Affiliations:** 1 Guangxi Hualan Geotechnical Engineering Co., Ltd., Nanning 530001, China; chenshengjin@gxhlyt.cn (S.C.); wgytzj@163.com (Z.T.); 2 School of Civil Engineering and Architecture, Guangxi University, Nanning 530004, China; jie_jiang001@126.com

**Keywords:** stabilized soil, red mud, synergistic effect, nano-SiO_2_, strength characteristics, unconfined compressive strength, stabilized soil

## Abstract

Based on the existing research results, this research team developed roadbed stabilized soil materials using nano-SiO_2_ synergistically modified red mud in order to study whether the strength of the stabilized soil materials meets the strength requirements of the roadbed materials, and at the same time, analyze its strength characteristics to make the feasibility of it being used as a roadbed material clear. Through different combination schemes, the effects of different nano-SiO_2_ and cement contents on the strength of the stabilized materials were explored. The test results show the following: In the synergistic modification of nano-SiO_2_ and cement, nano-SiO_2_ can significantly improve the early unconfined compressive strength of red mud-based stabilized soil. In the synergistic modification of nano-SiO_2_, gypsum, and cement, the 7 d unconfined compressive strength of red mud-based stabilized soil is greater than 2 MPa, which meets the strength requirements of road base materials and shows the superiority of synergism. The nominal stress–strain curves are divided into five stages: compressed and compacted stage, elastic deformation stage, plastic deformation stage, damage deformation stage, and residual deformation stage. The macroscopic compressive damage pattern of the specimens shows that the modified red mud-based stabilized soil mostly exhibits brittle damage. Tests have shown that the strength of modified terracotta-based stabilized soil meets the requirements of roadbed strength.

## 1. Introduction

Red mud is a solid waste generated during alumina production [1,2], and its utilization rate is extremely low [3]. With the increase in alumina production capacity worldwide, large amounts of red mud are generated every year [4,5], and red mud requires large amounts of land for stockpiling, which has a huge impact on both the natural environment and land resources [6,7]. Reducing red mud piles and promoting the resourceful use of red mud are important issues affecting the development of the aluminum industry.

The key to solving environmental problems in red mud disposal is to develop technologies that can directly consume large quantities of red mud or convert it into a secondary resource for reuse. Currently, in the engineering field, red mud is mainly used in the manufacture of bricks and ceramics or as a raw material for road base material [8,9,10,11]. Ou et al. [12] blended red mud with bauxite tailings and cement with different mass ratios to develop new road base materials, and the results showed that the 7-day unconfined compressive strength (UCS) was 3.03 MPa at a ratio of red mud to bauxite tailings of 2:1, which met the strength requirements of low-grade roads of class II and below. Ma et al. [13] used red mud as an additive for loess, and the results showed that adding appropriate amounts of red mud will promote the production of hydration products and effectively improve the unconfined compressive strength and shear strength of the treated loess. Scholars [14,15,16,17] have shown that utilizing red mud to partially replace cement or concrete reduces the amount of cement used and lowers carbon emissions and that the material’s strength characteristics are similar to those of cement or cement mortar, with no significant reduction in strength. Ou et al. [18] developed foam lightweight soil by using red mud, bauxite tailings mud foam, and the UCS and water stability coefficient of the specimens were at maximum when the red mud dosing was 16%, but the dry and wet cyclic effects significantly reduced the unconfined compressive strength of the foam lightweight soil. Mukiza et al. [19] investigated the possibility of using red mud as road base material and soft subsoil stabilizer material. It was found that the performance of red mud as road base material was superior to that of natural soil, and the synergistic use of red mud and other wastes also improved the mechanical and durability properties of the material compared to the use of red mud alone. Red mud contains traces of radioactivity. Li et al. [20] found that the hydration of cement forms a dense solidified body to adsorb and encapsulate radionuclides in the red mud, thus realizing the shielding effect on radioactive rays. At the same time, the addition of water and the production of crystalline water in the hydration products dilute the radionuclide content and also play a role in reducing radioactivity. This literature shows clearly that scholars have promoted the application of red mud reduction by using red mud as a major or partial raw material in engineering practice. As a base material, red mud also shows great potential in road base filling. It is expected that the use of red mud-based materials in this field can greatly benefit the process of red mud reduction. However, when red mud is used as a base material, care should be taken to reduce its alkalinity or take good anti-seepage measures for it.

Nano-SiO_2_ has a high specific surface area and good volcanic ash activity in alkaline environments, which can accelerate the hydration of nano-SiO_2_-cement systems to form complex microstructured C-S-H gels to improve early strength [21,22,23,24]. Meanwhile, it was proved that Nano-SiO_2_ can play a role in the reuse of red mud. Ahmed et al. [25] added nano-SiO_2_ in the synthesis of a geopolymer binder with red mud as the main solid source and found that the addition of suitable levels of nano-SiO_2_ contributed to the formation of geopolymer gels and C-S-H, giving geopolymer higher compressive strength. The relevant literature shows that nano-SiO_2_ has obvious performance advantages and can play a great role in civil engineering. However, there is little research on how to utilize the advantages of nanomaterials in red mud modification for strength improvement. Meanwhile, whether the strength of the modified red mud-based stabilized soil meets the strength requirements of roadbed materials and strength characteristics needs to be further studied. Therefore, this paper aims to develop red mud-based stabilized soil materials and synergistically involves nano-SiO_2_ in the modification of red mud stabilized soil to study the effect of nano-SiO_2_ on the enhancement of the strength of red mud stabilized soil. The thesis focuses on analyzing the strength characteristics of modified red mud-based stabilized soil and studying the feasibility of using it as a road base material from the perspective of strength. The research results can provide a reference for the resource utilization of red mud.

## 2. Materials and Methods

### 2.1. Raw Materials

#### 2.1.1. Red Mud

The red mud used was Bayer red mud, taken from an aluminum company in Guangxi Province. The specific gravity of the tested red mud was 3.06. The natural moisture content of the red mud was in the range of 28.10~35.21%.

**Figure 1:**
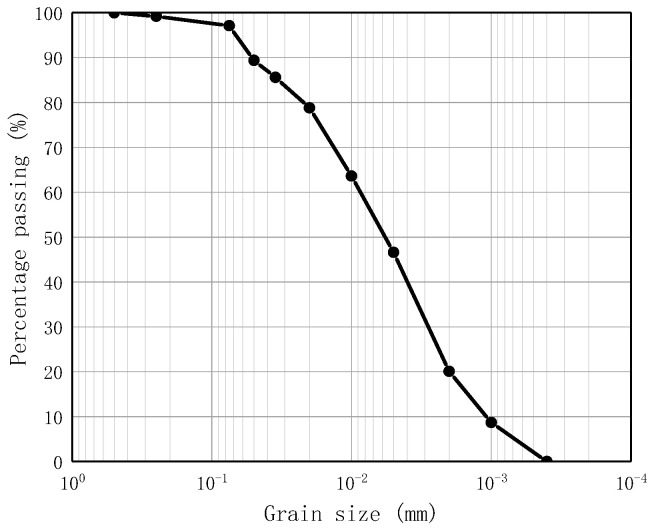
Particle gradation curve of the tested red mud.

In accordance with the standard for geotechnical testing method [26], the particle composition of red mud was tested by the densitometry method. The particle analysis curve of the tested red mud is shown in Figure 1. It shows that the content of particles with particle size less than 0.075 mm is 97.8%, while the content of particles with particle size less than 0.01 mm is 63.6%, which is categorized as clay according to the size of the particles. The test measured that the plastic limitof red mud was 0.75, and the liquid limit was 14. According to the Unified Soil Classification System, red mud belongs to low plasticity clay (CL).

The red mud contains trace heavy metal elements. In accordance with the Chinese industry’s standard solid waste extraction procedure for leaching toxicity—the acetic acid buffer solution method [27]—a leaching toxicity test for red mud was conducted. After testing, the leaching concentration of heavy metals in red mud is shown in Table 1.

Combined with the Chinese national standard for pollution control on the landfill site of municipal solid waste [28], it was determined that the leaching toxicity of heavy metals in the tested red mud did not exceed the standard.

#### 2.1.2. Nano-SiO_2_

Nano-SiO_2_ has a high specific surface area and good volcanic ash activity in alkaline environments, which improves early strength. The selected nano-SiO_2_ was produced by the mechanical crushing process. The nano-SiO_2_ is a white spherical powder with 99.9% purity. Its particle size ranges between 1 and 100 nm. The specific surface area of the nano-SiO_2_ particle is 240 m^2^/g.

#### 2.1.3. Cement

Cement is a commonly used curing agent that significantly increases the strength of modified soils. The cement used for the test was 42.5-grade ordinary silicate cement with a specific surface area of 340 m^2^/kg and a density of 3.10 g/cm^3^. According to the Standard Specification for Portland Cement [29], the selected cement type is Type I.

#### 2.1.4. Gypsum

Gypsum can continuously provide Ca^2+^ ions to the liquid phase of red mud-based stabilized soil. Under the alkaline environment, the secondary hydration reaction occurs, generating C-S-H and C-A-H hydrated gels, which enhance the strength of the modified soil. The gypsum chosen for the experiment is a kind of construction gypsum. It is a white powder with a density of 2.32 g/cm^3^, and its chemical formula is CaSO_4_-0.5H_2_O.

### 2.2. Experimental Design

In combination with the studies of [21,30], nano-SiO_2_ was incorporated into the red mud-based stabilized soil at a mass ratio of 0.5%, 1%, 1.5%, 2%, 2.5%, and 3%; gypsum was incorporated into the red mud-based stabilized soil at 6% of mass ratio; and cement was incorporated into the red mud-based stabilized soil at 1%, 3%, 5%, 7%, and 9% by mass.

The test procedure was as follows:

1. Adding cement alone to dry red mud and testing the effect of the individual admixture of cement on the strength of the specimen;
2. The nano-SiO_2_ dosing of 1% was selected to test the effect of different cement dosing on the unconfined compressive strength of specimens under the synergistic effect of nano-SiO_2_;
3. The optimum dose of 3% cement and 6% gypsum was selected from both strength and economic considerations, and different mass ratios of nano-SiO_2_ were added on this basis to test the synergistic effects of the three modified materials on strength control and to determine the optimum nano-SiO_2_ dosage.

The synergistic combination scheme of the experimental design is shown in Table 2, where RM is red mud; NS, PC, and CS are the abbreviations of nano-SiO_2_, cement, and gypsum, respectively; the following number refers to the blending mass ratios of corresponding materials (%).

### 2.3. Specimen Preparation

**Figure 2:**
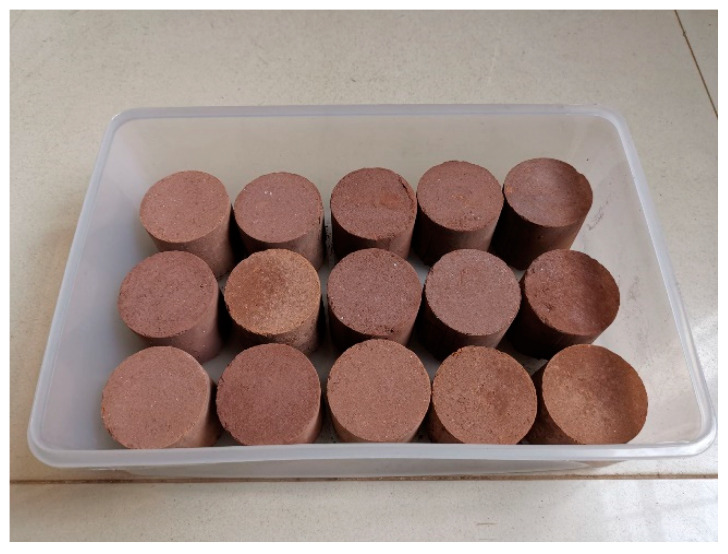
Photographs of cylindrical specimens.

The stabilized soil with different sterilizers, as shown in Table 1, was compacted into cylindrical specimens of 50 mm × 50 mm (Figure 2) as per the Test Method of Materials Stabilized with Inorganic Binders for Highway Engineering [31] and Liu et al. [32].

The specimen preparation and curing steps are as follows:

1. Firstly, the sampling red mud was dried at room temperature and crushed into powder with a plastic hammer; then, the crushed powder was sieved using a 0.5 mm steel sieve; the sieved red mud was oven-dried at 105 °C to further remove the moisture.
2. In accordance with the optimal water content determined by the compaction test, the modified materials (stabilizers, all in powder form) and a certain mass of deionized water were added into the red mud according to the designed dosage and mixed thoroughly.
3. The stabilized soil was compacted to a 5 mm × 5 mm cylindrical column. Note that the compaction should be done within 1 h after adding cement.
4. The compacted specimens were sealed with plastic film and placed in an environmentally controlled chamber with a temperature of 20 °C and relative humidity of 95° for curing. After reaching different curing periods of 1, 7, 14, 28, 60, and 120 days, the specimens were taken out for unconfined compressive strength tests.

### 2.4. Unconfined Compressive Strength Test

The unconfined compression test was carried out by a TSZ series fully automatic triaxial instrument produced by Nanjing Soil Instrument Factory Co. (Nanjing, China). The instrument can directly obtain the experimental maximum compressive strength and the relationship curve between the main stress difference and axial strain. The experimental parameters were set as follows: specimen height, 5 cm; specimen diameter, 5 cm; rigid steel ring coefficient, 30 N/mm; axial strain, 15%; loading level, 1 level; sampling step, 0.125 mm; shear rate, 2.5 mm/min.

**Figure 3:**
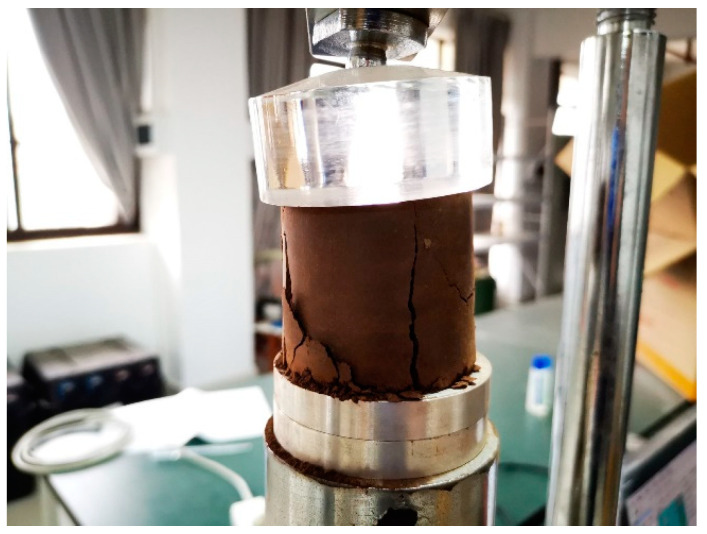
Specimen damage by compression.

Once the experiment has started, the instrument can automatically collect the compressive strength data and generate the stress–strain curve simultaneously until the specimen is completely damaged and loses its compressive capacity. Figure 3 shows the damaged state of the tested specimen.

The unconfined compressive strength is calculated according to Equation (1):

(1) $$q_{u}=\frac{P}{A}$$

where *q_u_* is the compressive strength of the specimen (MPa), accurate to 0.01 MPa; *P* is the breaking load of the specimen (N); *A* is the pressure-bearing area of the specimen (mm^2^).

## 3. Results and Analysis

### 3.1. Effect of Cement

**Figure 4:**
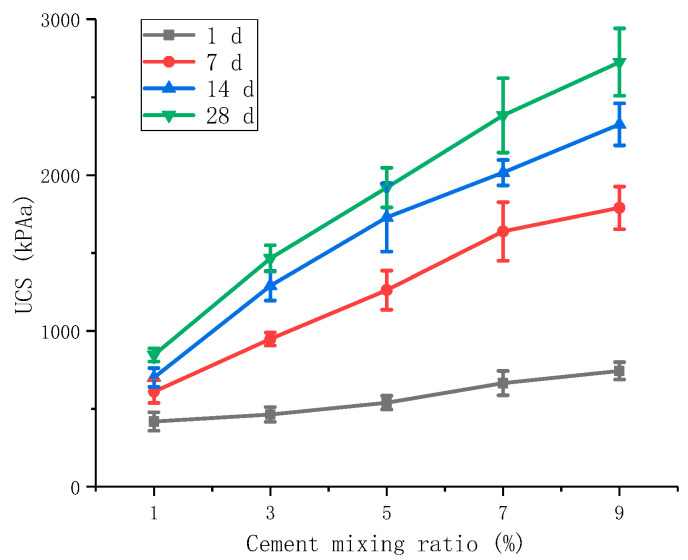
Effect of cement on UCS.

When the cement was modified alone, the specimens were dosed with 1%, 3%, 5%, 7%, and 9% of cement. The test results are shown in Figure 4.

Figure 4 shows that the unconfined compressive strength at 7 d is 491 kPa, 928 kPa, 1262 kPa, 1639 kPa, and 1797 kPa for 1%, 3%, 5%, 7%, and 9% of cement admixture, respectively. The unconfined compressive strength increases to 437 kPa, 334 kPa, 377 kPa, and 152 kPa for each 2% increase in cement, indicating that the increase in cement admixture can lead to higher unconfined compressive strength of the red mud-based stabilized soil.

The unconfined compressive strength of cement-modified red mud-based stabilized soil increased with the increase in the curing time. The unconfined compressive strengths of cement with 3% admixture are 463, 948, 1289, and 1466 kPa at 1, 7, 14, and 28 d, respectively, with the growth values of the first 7 and 28 d compressive strengths accounting for 48.3% and 82.3% of the growth values of the 28 d compressive strength.

### 3.2. Effect of Synergistic Modification

Management scientist Ansoff [33] believes that synergy is a system in which subsystems or elements cooperate, through the relationship of mutual collaboration, to achieve the overall synergy of the core content to obtain the effect of “1 + 1 ≥ 2”, resulting in the effect of “the whole is greater than the sum of the parts “. In this section, the characteristics of synergistic modification of nano-SiO_2_, gypsum, and cement will be analyzed, focusing on the effect of the synergistic effect of nano-SiO_2_ on the strength of red mud-based stabilized soil.

#### 3.2.1. Effect of Nano-SiO_2_ and Cement Co-Modification

**Figure 5:**
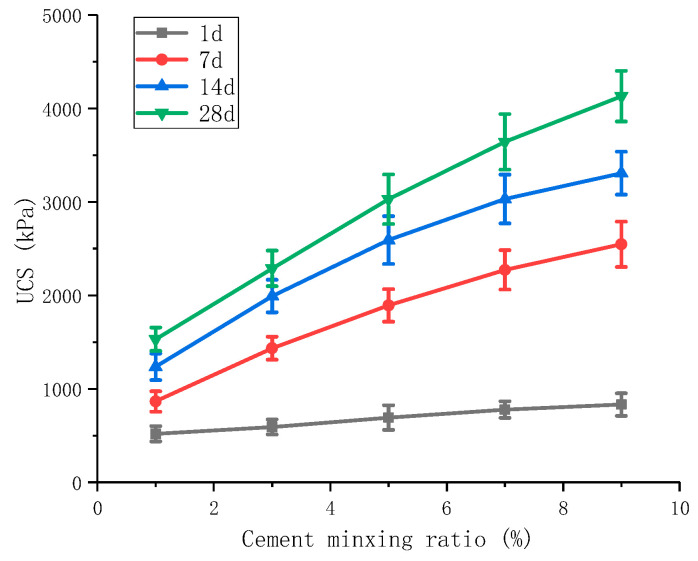
Effect of nano-SiO_2_ and cement on UCS.

**Figure 6:**
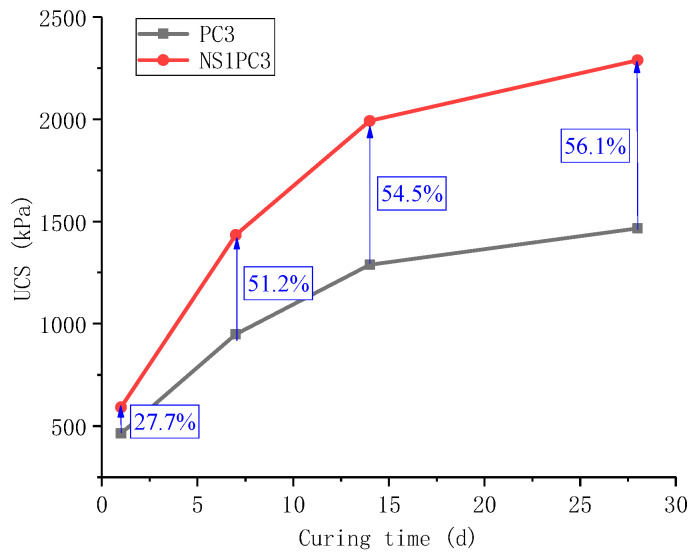
Comparison of UCS of NS1PC3 and PC3.

The increase in the unconfined compressive strength of the red mud-based stabilized soil was tested by adding 1% nano-SiO_2_ with the cement admixture still at 1%, 3%, 5%, 7%, and 9% and the nano-SiO_2_ in cooperation with the cement. The test results are shown in Figure 5 and Figure 6.

Figure 5 shows that the unconfined compressive strength of the red mud-based stabilized soil modified by nano-SiO_2_ co-cement showed a linear increase with the increase in cement admixture. For example, the unconfined compressive strength at 7 d was 865, 1434, 1892, 2273, and 2547 kPa for 1%, 3%, 5%, 7%, and 9% of cement admixture, respectively. For each 2% increase in cement, the increases were 569, 458, 381, and 274 kPa, respectively.

The unconfined compressive strength of the red mud-based stabilized soil modified with nano-SiO_2_ co-cement increased with the increase in the curing time, but the growth rate of the unconfined compressive strength was significantly greater when the curing time increased from 1 d to 7 d than when the curing time increased from 7 d to 14 d and from 14 d to 28 d. The growth rate of the unconfined compressive strength of the red mud-based stabilized soil modified with nano-SiO_2_ co-cement increased with the increase in the curing time.

Figure 6 shows the comparison of unconfined compressive strength with curing time for the addition of 1% nano-SiO_2_ with and without the addition of nano-SiO_2_, provided that the cement admixture is 3%. The NS1PC3 combination of nano-SiO_2_ synergistically modified with cement showed a large increase in unconfined compressive strength with curing time. For the same curing age, the unconfined compressive strength of the NS1PC3 synergistically modified combination increased by 27.7%, 51.2%, 54.5%, and 56.1%, respectively, compared to the PC3 alone modified combination. In addition to this, there is a characteristic that the growth of the lateral limit compressive strength tends to increase from 1 to 7 d, and the subsequent growth is more moderate, indicating that the growth rate of the lateral limit compressive strength is mainly concentrated from 1 to 7 d. This indicates that the synergistic effect of nano-SiO_2_ and cement improves the early unconfined compressive strength of the red mud-based stabilized soil.

#### 3.2.2. Effect of Nano-SiO_2_ Co-Modification with Cement and Gypsum

**Figure 7:**
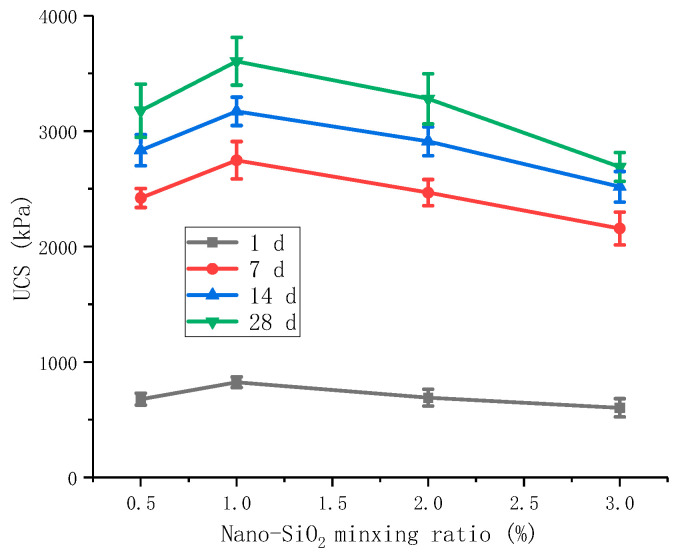
Effect of nano-SiO_2_ doping on the lateral limit compressive strength of the synergistic combination of gypsum and cement.

Under the premise of 3% of cement and 6% of gypsum, nano-SiO_2_ was selected at 0.5%, 1%, 2%, and 3% to test the increase in unconfined compressive strength of red mud-based stabilized soil under the action of nano-SiO_2_ in cooperation with cement and gypsum. The test results are shown in Figure 7.

It can be found that when the curing time was 7 d, the unconfined compressive strengths of nano-SiO_2_ with 0.5%, 1%, 2%, and 3% doping were 2421, 2748, 2467, and 2156 kPa, respectively. The unconfined compressive strength did not increase with the increase in nano-SiO_2_ doping, and the highest strength was achieved with the combination of nano-SiO_2_ with 1% doping. Referring to the Technical Guidelines for Construction of Highway Roadbases [34], the 7 d unconfined compressive strength of red mud-based stabilized soil is required to reach 2 MPa.

In contrast, the unconfined compressive strength of the modified red mud-based stabilized soil increased with the increase in the curing time, and the growth rate of the unconfined compressive strength was the largest in the curing age of 7 d. The growth value of compressive strength in the first 7 d accounted for 69.2% of the growth value of compressive strength at 28 d, based on the nano-SiO_2_ dosing of 1%. Figure 7 also shows that the highest unconfined compressive strength was obtained for the combination of gypsum and cement involved in the synergistic modification at 1% nano-SiO_2_. The 7 d unconfined compressive strength of NS1CS6PC3 reached 2748 kPa, which meets the strength requirements as a general road stabilized soil. The cement dosing was not the highest for the selected combination, considering the economic requirements, which is the optimal combination from the strength point of view. The next best combination is NS2CS6PC3.

#### 3.2.3. Analysis of the Synergistic Effect of Nano-SiO_2_

In this section, the synergistic modification characteristics of nano-SiO_2_, gypsum, and cement will be analyzed, focusing on the synergistic effect of nano-SiO_2_.

The 7 d unconfined compressive strength results of the gypsum and cement dosing for the optimal combination determined in the test, i.e., at 6% gypsum and 3% cement, for the corresponding combinations with and without nano-SiO_2_, were tabulated and analyzed as shown in Table 3.

The analysis of the synergistic effects of the involvement of nano-SiO_2_ before and after modification concluded the following:

(1) When the red mud is unmodified, the unconfined compressive strength of the specimens made under the conditions of 93% compaction, optimum moisture content, and maximum dry density is 388 kPa, which has a structural strength. However, because no modified material is added, the unconfined compressive strength cannot be enhanced with the increase in curing time. At the same time, the structure of the compacted specimens will be damaged if disturbed by external forces.
(2) Cement modification alone can make the red mud-based stabilized soil obtain higher compressive strength than that of unmodified, and the 7 d unconfined compressive strength with 3% cement admixture is 948 kPa, which is about 2.5 times that of unmodified compressive specimens. When 1% nano-SiO_2_ was added, the compressive strength of the red mud-based stabilized soil reached 1434 kPa, which was 1.5 times that of the compressive strength of the red mud-based stabilized soil modified by PC3 alone. The synergistic effect of nano-SiO_2_ promoted the modification of the red mud-based stabilized soil and led to further growth of the lateral-free compressive strength.
(3) When nano-SiO_2_ was modified with CS6 and PC3, the modified red mud-based stabilized soil obtained higher unconfined compressive strength. The 7 d unconfined compressive strength obtained at 1% nano-SiO_2_ was 2748 kPa, which was greater than the strength of 591 kPa of NS1 modified with CS6 and 1434 kPa of NS1 modified with PC3, and greater than the sum of 2025 kPa. This value is greater than the strength of NS1-modified soil with CS6 of 591 kPa and the strength of NS1-modified soil with PC3 of 1434 kPa and is greater than the sum of both of them of 2025 kPa. The synergistic effect of nano-SiO_2_ with CS and PC has obtained the effect of “1 + 1 > 2”.
(4) The above series of unconfined compressive strength tests gradually revealed the optimal amount of the three modified materials while further revealing the superiority of the synergistic effect to enhance the compressive strength of the modified materials, and NS1CS6PC3 was the best modified solution for the test from both economic and strength considerations.

### 3.3. Compressive Damage Characteristics of Red Mud-Based Stabilized Soil

#### 3.3.1. Typical Damage Characteristics

**Figure 8:**
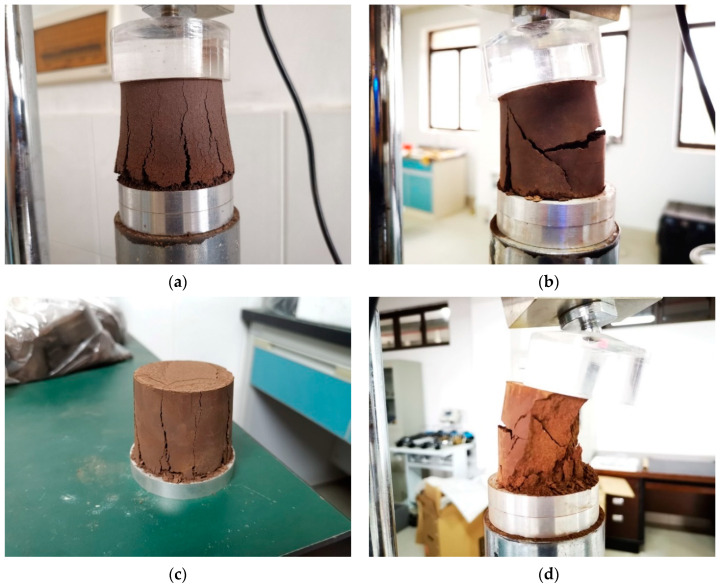
Typical damage pattern of red mud-based stabilized soil under pressure. (**a**) Typical damage pattern of red mud-based stabilized soil without modified materials under pressure. (**b**) Typical damage pattern of red mud-based stabilized soil with modified materials under pressure. (**c**) Lateral and top surface morphology of red mud-based stabilized soil with modified materials after compression damage. (**d**) Internal morphology of red mud-based stabilized soil with modified materials after compression damage.

Figure 8 shows the typical damage pattern of the red mud-based stabilized soil specimen under compression.

Figure 8a shows the compression damage pattern of the pure red clay-based stabilized soil, which shows the typical plastic damage under the uniaxial compression condition. The cracks are continuously developed upward and finally penetrate to the top of the specimen until the specimen is completely destroyed, and the bottom bulge also causes the cracks to be wide at the bottom and thin at the top.

Figure 8b shows the typical damage pattern of red clay-based stabilized soil under compression after the addition of modified materials. Most of the modified red clay-based stabilized soil shows brittle damage, especially when the modified materials and the curing age promote the increase in nominal stress; the brittle damage characteristics are more obvious. Under the load, the specimens did not show a bulging phenomenon. Under the condition of increasing uniaxial pressure, the specimens began to show fine cracks along the direction of loading; the cracks also started to appear from the bottom but would penetrate quickly from the bottom to the top (Figure 8c). Local specimens would show oblique cracks, but vertical cracks were dominant. In the stage of damage deformation, the cracks keep increasing, local slip appears at the specimen bottom, and the penetration cracks cut the specimen into several pieces. At the same time, the outer layer of the specimen appears to fall off due to cracking. When the strain increases, transverse cracks appear on parts of the side, and the transverse cracks promote shear damage to the side. When the side without transverse shear damage is also damaged, the specimen enters the final sharp decay of the stress, and finally, the stress tends to zero due to the complete destruction of the specimen (Figure 8d).

#### 3.3.2. Nominal Stress–strain Relationship

**Figure 9:**
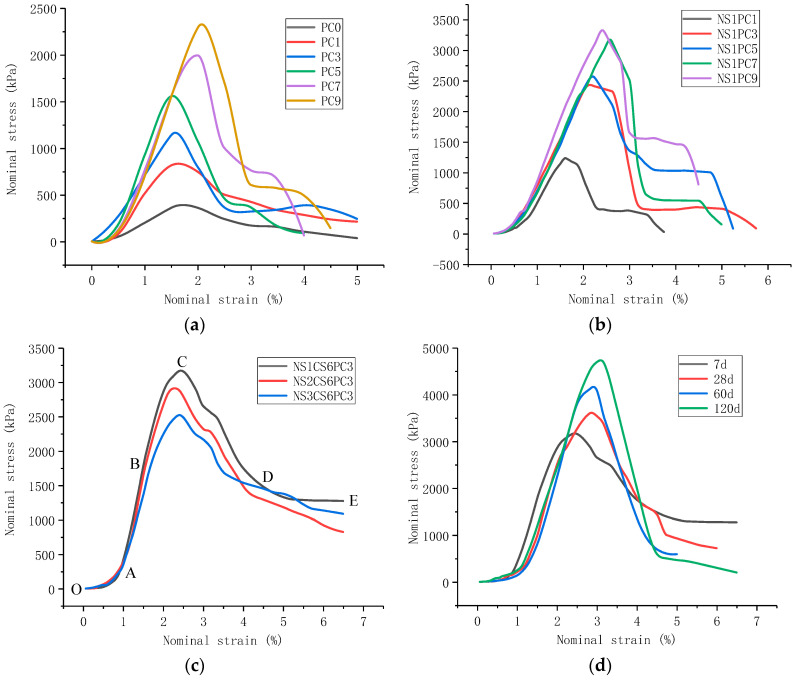
Nominal stress–strain relationship curve of modified red mud-based stabilized soil. (**a**) Nominal stress–strain curve of cement-modified specimens at 28 d age. (**b**) Nominal stress–strain curves of specimens modified with SiO_2_ and cement at 28 d. (**c**) Nominal stress–strain curves of specimens modified with nano-SiO_2_, gypsum, and cement at the age of 28 d. (**d**) Nominal stress–strain curve of NS1CS6PC3 modified combination with curing time.

Figure 9 shows the nominal stress–strain curves of the red mud-based stabilized soil under compression for each modification condition.

Figure 9 shows that the nominal stress–strain curves of the red mud-based stabilized soil under compression under each modification condition exhibit elastic-plastic deformation, which can be roughly divided into five stages according to the curve morphology. The curve morphology of each stage varies under different modification conditions. The stress–strain curves of the combined specimens of nano-SiO_2_, gypsum, and cement synergistically modified at the age of 28 d are used to label the stage division.

In the first stage, the OA section, the slope of the stress–strain curve increases continuously and is concave, but the course is short. The analysis suggests that the specimen is formed in this stage because the pore ratio under pressure becomes smaller, the specimen soil particles and the modified materials not involved in the reaction are further compacted, and the stiffness increases, resulting in increasing compressive strength. This stage belongs to the “compacted stage”, and the shape of the curve of the red mud-based stabilized soil is basically the same in this stage under each modification condition.

In the second stage, the AB section, the stress–strain relationship curve in this stage is approximately linear, and the nominal stress of the red clay-based stabilized soil increases linearly with the increase in the nominal strain. This stage is the “elastic deformation stage”, and the stress corresponding to the B point is the proportional limit. The shape of the second stage is slightly different under different modification conditions, but in general, the higher the compressive strength is, the higher the B point is in the stress rise stage. As shown in Figure 8a,b, the higher the PC doping, the higher the B point. Figure 8c shows the highest B point at 1% nano-SiO_2_, while Figure 8d shows that the higher the age of curing, the higher the B point. In the elastic deformation stage, the red clay-based stabilized soil resists the load through the cohesion and friction between the material particles, and the nominal strain and stress further increase with the progressive increase in load one. However, it has not yet exceeded the material resistance, so the deformation cracks have not yet appeared in the specimens at this stage.

In the third stage, the BC section, the slope of the stress–strain curve in this section is decreasing, the shape of the curve is concave, the stress reaches the peak and then stops growing, and the peak stress, i.e., the corresponding stress at point C is the unconfined compressive strength of the test material. This stage is the “plastic deformation stage” of the test material, which is also the “yielding stage”. In this stage, after the stress exceeds the proportional limit, the strain increases significantly with the increase in stress, and the terracotta stabilized soil softens, resulting in microcracks in the test material, which are mainly parallel to the loading direction. Then, with a further increase in load, the microcracks in the specimen also increase and gradually extend and expand, and the duration of this process is relatively short. The presence of penetrating microcracks implies irreversible damage to the red mud-based stabilized soil. However, the curve pattern shows that the specimen material can still withstand the increased load until the stress reaches the peak. When the stress state reaches the peak, a fall occurs, which is caused by the continuous development of microcracks through the development of the material load-bearing capacity decreases and destabilization, and finally enters the fourth stage—the damage deformation stage.

In the fourth stage, the CD section, after the curve crosses the peak from point C, the nominal stress decreases sharply with the increase in strain; this stage is the “damage deformation stage” of the material. Also similar to the second stage, the curve shape is slightly different under different modification conditions. In general, the higher the strength of the material, the more rapidly the stress decay, such as PC alone. For example, the higher the PC dose, the higher the rate of decline of the curve for the red clay-based stabilized soil modified by PC alone, and the greater the rate of decline of the curve for the red clay-based stabilized soil modified by NS1CS6PC3, the greater the rate of decline of the curve at this stage with the increase in the curing age, showing the characteristics of “brittle damage”. In the “damage deformation stage” of the material, the cracks of the red clay-based stabilized soil penetrate the top and bottom of the specimen; the cracks keep increasing, and the material strain of the specimen softens and destabilizes, leading to the rapid decay of the material resistance.

The fifth stage, the DE section, is the “residual deformation stage” of the test material. In this stage, the specimens produce a lot of plastic deformation. Terracotta-based stabilized soil, after the destruction of the stress, does not disappear completely, and even some specimens still retain a small value; the strength is the residual strength, but with the increase in stress, the nominal stress will eventually tend to zero. The compressive specimens of each modified red mud-based stabilized soil also roughly show two different characteristics at this stage. Most of them have a stress “damage platform”, and after the “damage platform”, the nominal stress decays rapidly again, and a few of them do not have a “damage platform”, and the stress gradually decays. For example, in the case of the red mud-based stabilized soil specimen modified by cement alone, the higher the PC admixture, the higher the unconfined compressive strength. Furthermore, the “damage platform” is shown in the fifth stage, where the nominal stress does not continue to decay but appears to “stagnate” with strain growth; the nominal stresses decayed rapidly again and tended to zero until the end of the test. The stress–strain curves of NS1PC-modified red clay-based stabilized soil showed the characteristics of “damage platform” in all specimens at this stage, with the difference that NS1CS6PC3-modified red mud-based stabilized soil, the nominal stress did not show the platform effect when the curing age was short, but when the curing age reached 120 d, the platform effect was obvious. Combined with the analysis of the typical damage pattern of red mud-based stabilized soil under compression in Section 3.1, the modified red mud-based stabilized soil from the appearance of transverse cracks on one side to the appearance of transverse cracks on the other side of the period, the material resistance is borne by the side of the undamaged side. At this time, the growth of stress is very small, but the strain is still increasing slowly. This is the “damage platform” in the stress–strain curve, which is reflected in the macroscopic compressive pattern.

The ratio of residual stress to peak stress is shown in Table 4. The ratio of residual stress to peak stress decreases with the growth of the curing age, showing the development from plastic to elastic.

From the above analysis, it can be seen that the red mud-based stabilized soil has different deformation mechanisms during uniaxial compression. During the compressive compacting stage, the material mainly shows that the pore ratio becomes smaller, the material particles are further compacted, and the compressive strength is further enhanced. In the elastic stage, the material particle compactness continues to be strengthened, and the external load is resisted through the cohesion and friction between particles. When the external load is continuously increased, the resistance of the material is continuously enhanced, but still, no cracks appear to form in the material. In the plastic stage, microcracks appear in the material, gradually extend and expand, penetration cracks form and irreversible damage occurs. However, the material is not destabilized and damaged, and the compressive strength can still increase with the increase in external load and reaches the peak before the material destabilization and damage. Moreover, there is an obvious strain softening behavior of the material. In the damage stage, the cracks keep increasing and are accompanied by the specimen fragment body falling off from the main body of the specimen. The material resistance decays rapidly, but before the compressive capacity completely disappears. Before the complete loss of compressive capacity, most of the specimens showed the ductility characteristics of increasing stress invariant stress, indicating that the red mud-based stabilized soil has good buffering performance and still has a certain bearing capacity after the damage. Finally, with the increase in compressive time, the specimens were completely destabilized and destroyed.

## 4. Conclusions

In this experiment, the stabilized soil material was prepared by modifying the red mud from aluminum industrial waste, and the strength characteristics of the red mud-based stabilized soil with different modified materials and different amounts of modified materials were tested and analyzed. The main findings were as follows:

(1) Cement alone can improve the unconfined compressive strength of red mud-based stabilized soil; when nano-SiO_2_ is modified with cement, nano-SiO_2_ can significantly improve the early unconfined compressive strength of red mud-based stabilized soil; when nano-SiO_2_, gypsum, and cement are modified with 6% gypsum and 3% cement, under the synergistic effect of nano-SiO_2_ (1%, 2%, and 3%, respectively), the 7 d unconfined compressive strength of the red mud-based stabilized soil was greater than 2 MPa. The maximum was achieved at 1% nano-SiO_2_, satisfying the strength requirements of the roadbed material. The synergistic results of the addition of nano-SiO_2_, gypsum, and cement showed the superiority of synergy. Compared with other modification research results, the amount of red mud in the red mud-based stabilized soil is relatively high, accounting for 90%, so the application of the research results in roadbed filling can consume a large amount of red mud and has a good development prospect.
(2) The compressive damage patterns of the specimens showed that most of the modified terracotta-based stabilized soils exhibited brittle damage, especially when the modified materials and the age of maintenance promoted conditions that increased the nominal stresses. Under the condition of increasing uniaxial pressure, vertical cracks appeared in the specimens first, and with the increase in cracks, slip appeared at the bottom. As the pressure increases, transverse cracks appear in the specimen, and the transverse cracks promote shear damage on one side of the specimen. When the other side without transverse shear damage also produces damage, the specimen is finally completely destroyed.
(3) The compressive deformation of the red mud-based stabilized soil shows the characteristics of elastic-plastic deformation, and the nominal stress–strain curves of the red mud-based stabilized soil under each modified condition are divided into five stages: “compressive compacting stage”, “elastic deformation and plastic deformation stage”, “damage deformation and residual deformation stage”, and so on. Most of the specimens showed “damage platform”. Most of the specimens showed a ductile character with small changes in stress but with an increase in stress variation before the resistance to compression was completely lost, indicating that the red mud-based stabilized soil has good buffering performance. It is more suitable to be applied as road base material for general roads.

## Figures and Tables

**Table 1:** Leaching concentration table of main heavy metals in red mud.

Projects	Cu	Zn	Pb	Cd	Ni	As	Cr
leaching concentration (mg/L)	0.27	0.02	0.01	0.10	0.12	0.17	0.09
mass concentration limit (mg/L)	40	100	0.25	0.15	0.50	0.30	4.5

**Table 2:** Collaborative combination scheme table.

Projects	Synergistic Solution Portfolio				
	Program 1	Program 2	Program 3	Program 4	Program 5
RM unmodified	RM				
PC individually modified	PC1	PC3	PC5	PC7	PC9
NS + PC synergistic modification	NS1PC1	NS1PC3	NS1PC5	NS1PC7	NS1PC9
NS + CS6 + PC3 synergistic modification	NS0.5CS6PC3	NS1CS6PC3	NS2CS6PC3	NS3CS6PC3	

**Table 3:** UCS of stabilized specimens containing nano-SiO_2_ (unit: kPa).

Projects	NS Doping							Remarks
	0	0.5%	1%	1.5%	2%	2.5%	3%	
RM unmodified	388							Control test
PC3 separately modified	948							
NS1 + PC3 synergistic modification			1434					
NS + CS6 + PC3 synergistic modification		2421	2748		2467		2156	

**Table 4:** The ratio of residual stress to peak stress.

Curing Period (d)	0	14	28	60	120
Residual stress/peak stress	0.53	0.44	0.24	0.16	0.13

## Data Availability

Some or all data, models, and code that support the findings of this study are available from the corresponding author upon reasonable request.
